# Toll-Like Receptor 4 and High-Mobility Group Box 1 Are Critical Mediators of Tissue Injury and Survival in a Mouse Model for Heatstroke

**DOI:** 10.1371/journal.pone.0044100

**Published:** 2012-09-04

**Authors:** Mohammed Dehbi, Taher Uzzaman, Engin Baturcam, Abdelmoneim Eldali, Wilhelmina Ventura, Abderrezak Bouchama

**Affiliations:** 1 Department of Comparative Medicine, King Faisal Specialist Hospital & Research Centre, Riyadh, Kingdom of Saudi Arabia; 2 Biostatistics, Epidemiology and Scientific Computing, King Faisal Specialist Hospital & Research Centre, Riyadh, Kingdom of Saudi Arabia; 3 Department of Experimental Medicine, King Abdullah International Medical Research Center, Riyadh, Kingdom of Saudi Arabia; Charité-University Medicine Berlin, Germany

## Abstract

The molecular mechanisms that initiate the inflammatory response in heatstroke and their relation with tissue injury and lethality are not fully elucidated. We examined whether endogenous ligands released by damaged/stressed cells such as high-mobility group box 1 (HMGB1) signaling through Toll-like receptor 4 (TLR4) may play a pathogenic role in heatstroke. Mutant TLR4-defective (C3H/HeJ) and wild type (C3H/HeOuJ) mice were subjected to heat stress in an environmental chamber pre-warmed at 43.5°C until their core temperature reached 42.7°C, which was taken as the onset of heatstroke. The animals were then allowed to recover passively at ambient temperature. A sham-heated group served as a control. Mutant mice displayed more histological liver damage and higher mortality compared with wild type mice (73% vs. 27%, respectively, P<0.001). Compared to wild type mice, mutant mice exhibited earlier plasma release of markers of systemic inflammation such as HMGB1 (206±105 vs. 63±21 ng/ml; *P* = 0.0018 and 209±100 vs. 46±32 ng/ml; *P*<0.0001), IL-6 (144±40 vs. 46±20 pg/ml; *P*<0.001 and 184±21 vs. 84±54 pg/ml; *P* = 0.04), and IL-1β (27±4 vs. 1.7±2.3 pg/ml; *P*<0.0001 at 1 hour). Both strains of mice displayed early release of HMGB1 into the circulation upstream of IL-1β and IL-6 responses which remained elevated up to 24 h. Specific inhibition of HMGB1 activity with DNA-binding A Box (600 µg/mouse) protected the mutant mice against the lethal effect of heat stress (60% A Box vs. 18% GST protein, *P* = 0.04). These findings suggest a protective role for the TLR4 in the host response to severe heat stress. They also suggest that HMGB1 is an early mediator of inflammation, tissue injury and lethality in heatstroke in the presence of defective TLR4 signaling.

## Introduction

Heatstroke is a leading cause of mortality and morbidity during heat waves [1], [2]. It is characterized by an increase of the body temperature to above 40°C in humans and clinical manifestations being similar to those of sepsis, such as systemic inflammation, disseminated intravascular coagulation, and multiple organ system dysfunction that may culminate in death [3]. The mechanisms of multiple organ system dysfunction and death are not fully understood but excessive activation of the host inflammatory and hemostatic responses, endothelial injury and apoptosis have been implicated [3], [4], [5], [6], [7], [8].

Clinical and experimental studies consistently demonstrated the presence of a systemic inflammatory response to heat stress that correlated with severity and outcome [5], [6], [7], [8], [9], [10], [11], [12]. Neutralizing studies in animal models of heatstroke have established that this systemic inflammation has a significant role but it remains unclear whether it is beneficial or deleterious. As shown previously, inhibition of the inflammatory response with administration of IL-1 receptor antagonist [8], anti-inflammatory steroids [12] or recombinant activated protein C [7], [10] prevented organ damage and improved survival while other studies using IL-6 and tumor necrosis factor (TNF) receptors knockout mice [9] or primate models [11] reported enhanced mortality. One of the reasons that might explain these conflicting findings is that the molecular mechanisms that initiate and propagate the inflammatory response to heat stress are not known, although earlier studies have suggested that lipopolysaccharide (LPS) leaking into the systemic circulation from heat damaged gut is the primary stimulus [3], [9].

Toll-like receptors (TLRs) are a family of at least 11 proteins that function as central mediators of the innate immune response to diverse pathogens as well as to endogenous molecules released by injured or dying cells [13], [14]. Among these pattern recognition receptors, TLR4 recognizes both pathogen-induced LPS and damage-associated molecular patterns and, thus, is implicated in the innate immune response to both infection and sterile injury [13], [14]. Activation of the TLR4 signaling pathway by its ligands initiates the inflammatory response through downstream signaling resulting in NF-κB activation and production of pro-inflammatory cytokines [13], [14]. Many studies have shown that TLR4 signaling plays an important role in the pathogenesis of various conditions including ischemia/reperfusion injury, trauma and hemorrhagic shock [15], [16], [17], [18]. The role of TLR4 in the host response to heat stress remains to be elucidated, particularly that modulation of the TLR4 signaling pathway seems to offer a promising therapeutic approach [19], [20].

Several endogenous danger signal molecules including the high-mobility group box 1 (HMGB1) protein, heat shock proteins, and degradation products of the extracellular matrix have been identified as the ligands for TLR4 [14], [19], [21]. HMGB1 is a nuclear nonhistone DNA-binding protein that has emerged as an essential mediator of TLR4 signaling in many infectious and non-infectious conditions [19]. Initially described as a late mediator of endotoxin-related lethality in mice, HMGB1 is now recognized also as an early mediator of inflammation and organ damage in sterile injury [19], [22]. More recently, HMGB-1 has been documented in the sera in a heatstroke rat model and its neutralization with recombinant thrombomodulin (TM/CD141) resulted in reduced inflammation, tissue injury and death, suggesting that HMGB-1 is implicated in the pathogenesis of heatstroke [23].

In the present study, we tested the hypothesis that endogenous ligand HMGB1 released by damaged/stressed cells signaling through TLR4 may play a pathogenic role in heatstroke. More precisely, to determine whether the full development of inflammation, tissue injury and lethality from heatstroke were dependent on TLR4 signaling and its endogenous ligand HMGB1, we compared the responses of C3H/HeJ mice subjected to severe heat stress with those of the genetically related C3H/HeOuJ mice. C3H/HeJ mice belong to a strain that expresses a phenotype analogous to that of TLR4 knockout mice due to a missense mutation in the TLR4 gene, hindering signal transduction including that of LPS [24].

## Materials and Methods

### Reagents

Isopropyl-β-D-thiogalactopyranoside (IPTG), glutathione and agarose were purchased from Sigma (Sigma, St. Louis, MO, USA). All restriction enzymes were purchased from New England Biolabs (New England Biolabs, Ipswich, MA, USA).

### Animals

All procedures involving experimental animals were approved by the institutional Animal Care and Use Committee of King Faisal Specialist Hospital and Research Center. The surgical procedures were performed by licensed veterinarians and after agreement from the Animal Care and Use Committee. Male and female, wild-type and mutant specific pathogen-free (SPF) adult mice (8–10 weeks old; 25–30 g weight) used in this study were purchased from Jackson Laboratories (Bar Harbor, Maine, USA). To control for estrus activity, female mice were housed in the absence of male mouse pheromones. Food and water were provided *ad libitum* under standard laboratory conditions (ambient temperature of 25°C, relative humidity of 55% and a stable circadian cycle of 12 h light and 12 h dark).

### Surgical Procedures

Mice were subjected to general anesthesia with Isoflurane inhalation (5% induction, 2.5% maintenance in 1.5 L/min O_2_). Surgical preparation consisted of shaving the abdominal hair and disinfecting the shaved area with betadine and alcohol. A 1 cm incision was made through the skin and abdominal muscle and then a free transmitter (model TA10TA-F20; Data Sciences International, MN, USA) was placed in the abdominal cavity for remote monitoring of core body temperature (Tc). The abdominal muscle and skin layers were closed with sutures. Immediately after surgery, the closed area was disinfected with Betadine and the animals were allowed to recover under regular monitoring for at least 2 weeks. The criteria for recovery at the end of the 2-weeks includes regain of pre-surgical weight and a steady circadian core temperature.

### Heatstroke Induction

Conscious unrestrained mutant and wild type mice with core temperature monitored by biotelemetry as described by Leon et al [25] were randomly assigned into sham-heated control or heat stressed groups (n = 6–10 mice per group). The heat stressed group was placed inside a temperature and humidity controlled chamber (Vena Engineering Corporation) pre-set at 43.5°C until their core temperature attains 42.7°C which was taken as a reference point of heatstroke onset (T0). Then, the animals were removed from the incubator and allowed to recover at ambient temperature. The animals were allowed *water ad libitum* throughout the experiment. Our experimental model diverged from the one reported in other study [25] by the selection of higher ambient temperature (43.5°C) and the availability of water throughout the experiment. The animals in the control group instrumented with free transmitter in the abdominal cavity were sham-heated in the same chamber pre-set at 25°C for the same period of time as the study group. All mice were weighed on a top-loading balance immediately before the start and at the end of heat stress. In order to reduce any potential stress-induced hyperthermia due to the novel environment, mice from both groups were acclimatized to the chamber for a period of 24 h at ambient temperature of 25°C before experimentation.

### Blood and Organ Sampling

Animals were subjected to general anesthesia with Isoflurane before heat stress (baseline represented by sham-heated animals), at the onset of heatstroke (T0) and at T0+0.5, +1, +4 and +24 hours and then processed for blood withdrawal by cardiac puncture using heparinized tubes. Plasma was separated by centrifugation at 1550×g for 15 min and stored in aliquots at −80°C until assayed. Tissue samples from liver were obtained from all animal groups at T0, T0+1, and +4 hour as well as the sham-heated group, snap frozen in liquid nitrogen and stored at −80°C until assayed. For histological analysis, tissues were fixed in buffered formalin solution for overnight and paraffin embedded. Sections were stained with Hematoxylin-Eosin (H&E) using standard protocol.

### Cytokine Measurements

The plasma levels of TNF-α, IL-1β, IL-6 and HMGB1 were determined by ELISA using commercially available ELISA kits according to the recommendations of the manufacturer (R & D Systems, Minneapolis, MN, USA) and (IBL International GmbH, Hamburg, Germany), respectively.

### Cloning, Expression and Purification of Full Length HMGB1 Protein and its A-Box Domain

cDNA corresponding to full length of HMGB1 protein was PCR-amplified from a human brain Quick-Clone cDNA library (Clontech, Palo Alto, CA, USA) by using the forward primer (5′- gga **AGATCT** ATGGGCAAAGGAGATCCTAAG -3′) in conjunction with the reverse primer (5′- acgc **GTCGAC** TTATTCATCATCATCATCTTCT -3′). A similar strategy was used to clone the A Box domain of HMGB1 protein by using the forward primer (5′- gga **AGATCT** ATGGGCAAAGGAGATCCTAAG -3′) in conjunction with the reverse primer (5′- acgc **GTCGAC** TTACTTCTTTTTTGTCTCCCCTTTG -3′). The resulting products were digested with *Bgl* II/Sal I and cloned into the unique *Bam*H I/*Sal* I sites of pGEX-6P1 Amersham Biosciences (GE Healthcare, Piscataway, NJ, USA) as a C-terminal fusion with GST-tag, sequence verified and used to transform *E. coli* strain BL-21 (Sigma, St. Louis, MO, USA).

### Expression and Purification of HMGB1

Clones harboring HMGB1 or A-Box domain of HMGB1 were induced with 1 mM Isopropyl β-D-1-thiogalactopyranoside (IPTG) for 3 h at 37°C and the purification was done by using the Glutathione Sepharose affinity column (Sigma, St. Louis, MO, USA). GST protein that was expressed and purified from *E. coli* transformed with pGEX-6P1 vector was used as control for experiments using GST-HMGB1 proteins. Eluted proteins were passed over a polymyxin B column (Pierce, Rockford, IL, USA) to remove any contaminating LPS and subsequently concentrated by Centricon YM-10 column (Milipore, Billerica, MA, USA). Protein purity and integrity were monitored by Coomassie blue staining after separation on SDS-PAGE and the concentration was determined by Bradford assay using γ-globulin as a standard (BioRad, Hercules, CA, USA). Samples were aliquoted, snap frozen on liquid nitrogen and stored at −80°C.

### Pre-treatment of Animals with Recombinant a Box Protein

Prior to heat stress challenge, mutant mice received a single intraperitoneal (i.p.) injection of 600 µg/mouse of either GST-A Box (n = 10) or GST control protein (n = 10) in a final volume of 400 µl of sterile PBS and then subjected to heat stress until their body temperature reached 42.7°C. The A Box dose was selected on the basis of its efficacy in improving survival in a mouse model of sepsis [26]. After heat stress, the animals were allowed to cool passively as described above and the survival rate was monitored for 72 h after heatstroke onset. Animals that survived for more than 72 h were considered as permanent survivors.

### Thermal Calculation

Heat stress was quantified by determining the heat load, a product of magnitude of core temperature (T) above 39.5°C and duration of hyperthermia as described previously [25]. The temperature threshold of 39.5°C degrees represents a baseline above which heat stress occurs in mice. Core body temperature was recorded every 2-minutes, and heat load (°C-min.) was calculated as Σ time interval (min.)×0.5 [T (°C) above 39.5°C–39.5°C)]. Heating rate (°C/min.) was calculated as [T0 (42.7°C) attained during heat exposure – T (°C) recorded before heat exposure]/time (min.) to attain T0.

### Statistical Analysis

The data are presented as mean (±SD) for each group, unless indicated otherwise. Significance of differences between groups was determined by repeated measures analysis using the generalized estimating equations model. For continuous variables, groups were compared using Mann-Whitney test. Kaplan-Meier curves and log-rank test were used for survival analysis. Differences were considered statistically significant at the *P*-values <0.05. The statistical analysis of data was performed using the software package SAS version 9.2, (Statistical Analysis System, SAS Institute Inc., Cary, NC, USA).

## Results

### Establishment of Heatstroke

**Figure 1:**
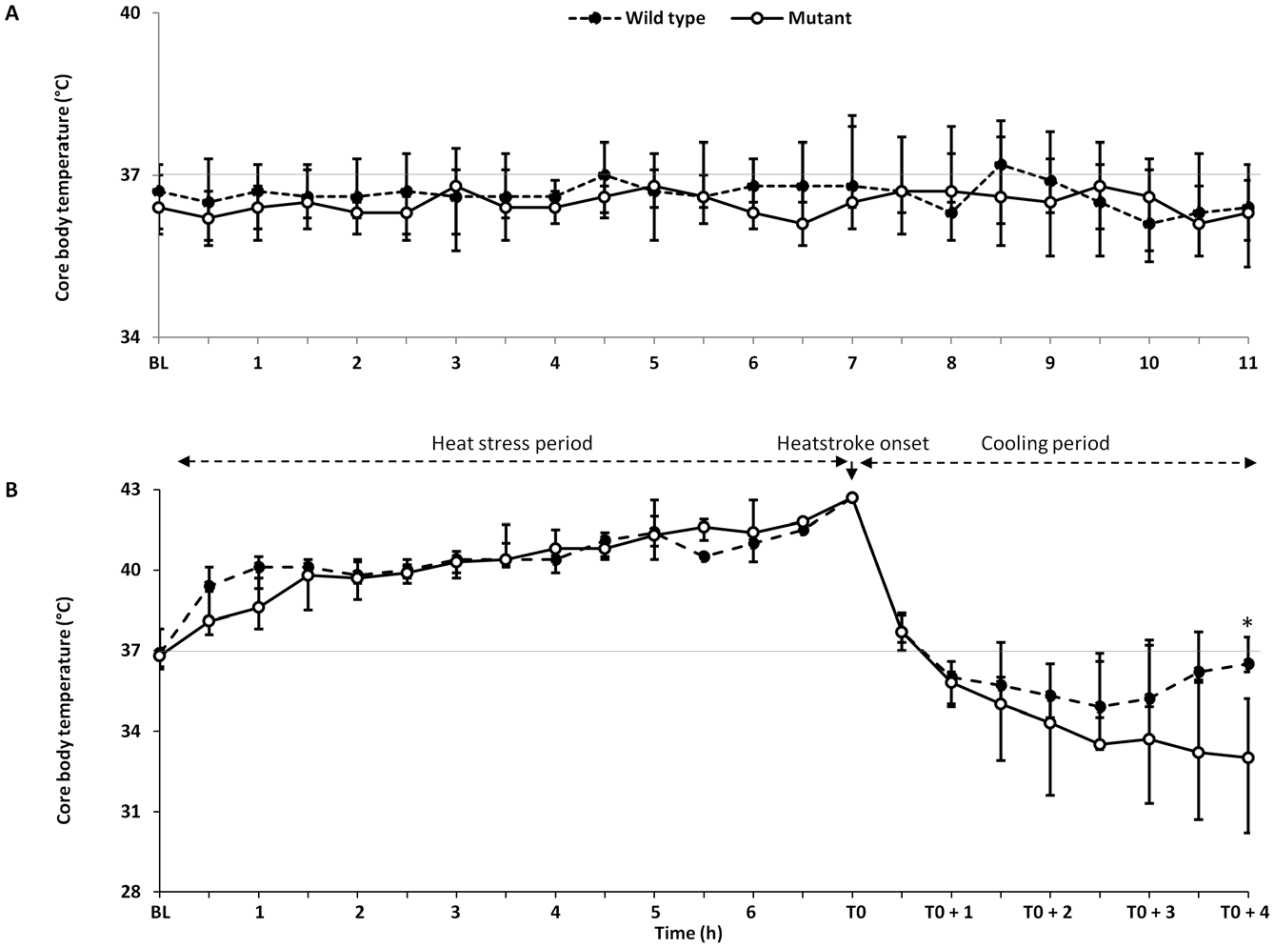
Core body temperature response of mutant and wild mice type to environmental heat stress. Mice implanted intraperitoneally with radiotelemetric transmitters (Data Science International were allowed to recover for 2 weeks after surgery before subjected to heat stress or sham heat stress. (**A**) Core body temperature recorded in mutant and wild type mice at baseline (BL), and during sham heat exposure. (**B**) Core body temperature recorded in mutant and wild type mice at baseline, during cooling and recovery after heatstroke. Values represent median and IQR. *P<0.05 at T0+4 hours between the two groups tested by the generalized estimating equations model.

To induce heatstroke, mutant and wild type mice implanted intraperitoneally with radiotelemetric transmitters were subjected to comparable heat stress as indicated by the maximum body temperature and the heat load (Table 1). When mice attained 42.7°C considered as the onset of heatstroke, they were removed from the incubator and allowed to recover passively at ambient temperature of 25–29°C. Although, water was allowed ad libitum throughout the experiment, heat stress induced body weight loss (around 10%) in all animals, with no significant differences between mutant and wild type mice. The thermoregulatory responses during the period of heat stress as well as cooling and recovery period differed significantly between mutant and wild type mice (Table 1 and Fig. 1B). The mutant mice displayed more resistance to heat stress as suggested by a significantly longer duration of heat exposure to reach their end-point core temperature than the wild type mice (Table 1). Also, the mutant mice displayed more profound and sustained hypothermia compared with wild type mice (Fig. 1B). In contrast, Figure 1B shows that the wild type mice exhibit a biphasic thermoregulatory responses namely an initial transitory hypothermia followed by an elevation of core temperature returning toward normal values consistent with the pattern described by Leon et al. [27]. The thermoregulatory response during sham-heat stress period was comparable between mutant and wild type groups (Fig. 1A).

**Table 1:** Thermal responses in Mutant and Wild-type mice subjected to heat stress.

Heat Response	Mutant	Wild-type	*P* value
Pre-heat stress Weight	27.8 (25.4–29.7)	27.5 (25.4–29.6)	0.61
Post-heat stress Weight	25.0 (22.7–27.4)	24.4 (22.1–27.2)	0.38
*T0 (°C)	42.7 (42.7–42.8)	42.7 (42.7–42.7)	0.62
Time at >39.5°C (min)	120(86–207)	188(134–241)	*0.38*
Heat load (°C/min)	248(148–251)	216(147–242)	0.50
Heating rate (°C/min)	0.021(0.018–0.031)	0.017 (0.014–0.024)	0.28
Duration of heat stress (min)	287 (212–373)	243 (176–321)	0.01

All values are median (25 and 75^th^ interquartile range). Statistical comparisons were made by Mann-Whitney test between mutant and wild-type mice.

* = target core temperature that signals heatstroke onset.

### Heatstroke-induced Lethality is Significantly Altered in Mutant Mice

**Figure 2:**
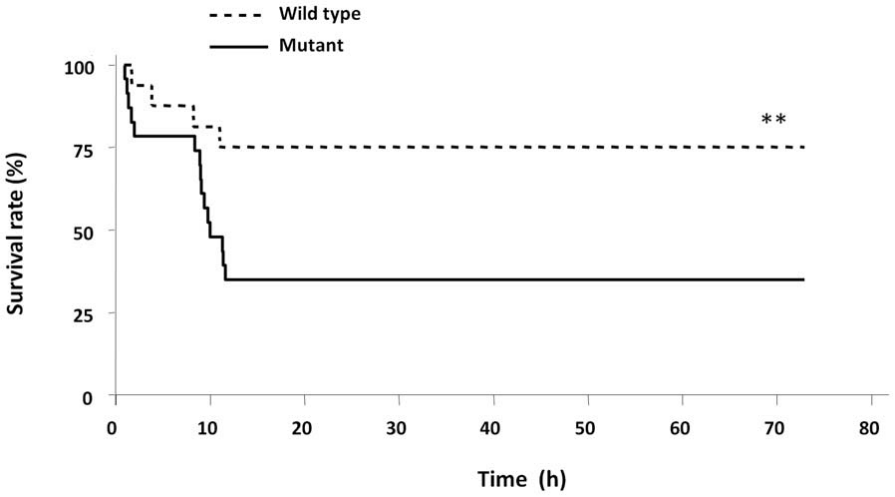
Comparison of heatstroke-induced lethality in mutant and wild type mice. Survival rates (%) of wild type and mutant mice after heatstroke are shown over 72 hours from the onset of heatstroke. Heatstroke was induced by passive exposure to environmental heat until the core temperature reached 42.7°C. **P<0.001 between the two groups analyzed by the Kaplan-Meier log-rank test.

To determine whether TLR4 was involved in the pathogenesis of heatstroke, the survival of mutant and wild type mice after heatstroke namely during cooling and recovery period was monitored. As shown in Fig. 2, the mortality rate was significantly higher for the mutant than the wild type strain (73% vs. 27%, respectively, *P*<0.001). In the sham-heated control group, all mice remained alive during the course of the experiment (data not shown).

### Heatstroke-induced Liver Damage is more Pronounced in Mutant Type Mice

**Figure 3:**
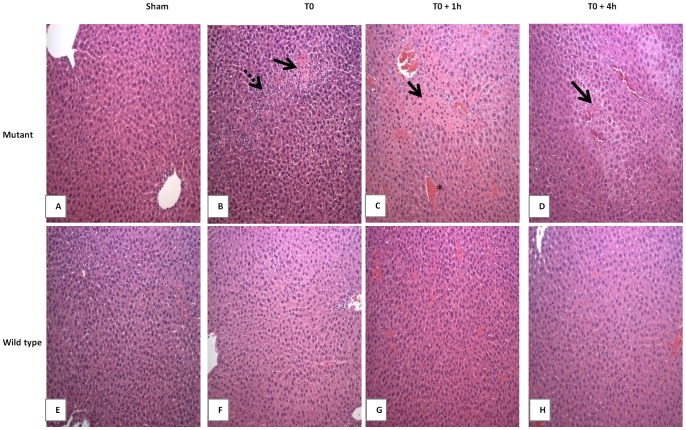
Histopathological comparison of heatstroke-induced liver injury in mutant and wild type mice. Heatstroke was induced in mice by passive exposure to environmental heat until the core temperature reached 42.7°C. Sham-heated mice were used as control. Liver sections from mutant (**A–D**) and wild type (**E**–**H**) mice either sham-heated (**A** and **E**) or after induction of heatstroke (**B–D** and **F–H,** respectively) were stained with Hematoxylin & Eosin. In both mice strains, no morbid changes were observed after sham-heat stress (**A** and **E**). Heatstroke-induced in mutant mice resulted in hepato-cellular necrosis (arrows) and inflammatory cell infiltration (broken arrow) at onset (**B**), and massive perilobular necrosis with severe sinusoidal congestion (*) were observed at follow-up (**C**, **D**) time points. Minimal histopathological changes were noted in wild type subjected to heatstroke (**F**–**H**).

To investigate whether the high mortality rate observed in mutant mice was consistent with organ injury, we examined the extent of histological liver damage in both strains at the onset of heatstroke, and during the recovery period. The liver, was selected because it is a vulnerable site to heat injury [28], [29]. As expected, there were no changes observed in the sham-heated group for both strains (Fig. 3A & E). In contrast, heat stressed animals displayed widespread damage that varied between the two strains. Extensive damage was observed in mutant mice following heat stress (Fig. 3B–D). The changes consisted of focal hepatocytes necrosis associated with marked inflammatory cells infiltration at the onset of heatstroke (Fig. 3B). At T0+1 and T0+4 hours following the onset of heatstroke, a massive perilobular hepatocytes necrosis and central vein congestion were observed (Fig. 3C–D). Minimal damage was noted in wild type mice (Fig. 3F–G).

### Heatstroke-induced an Early Inflammatory Response with Marked HMGB1 Release in Mutant Mice

**Figure 4:**
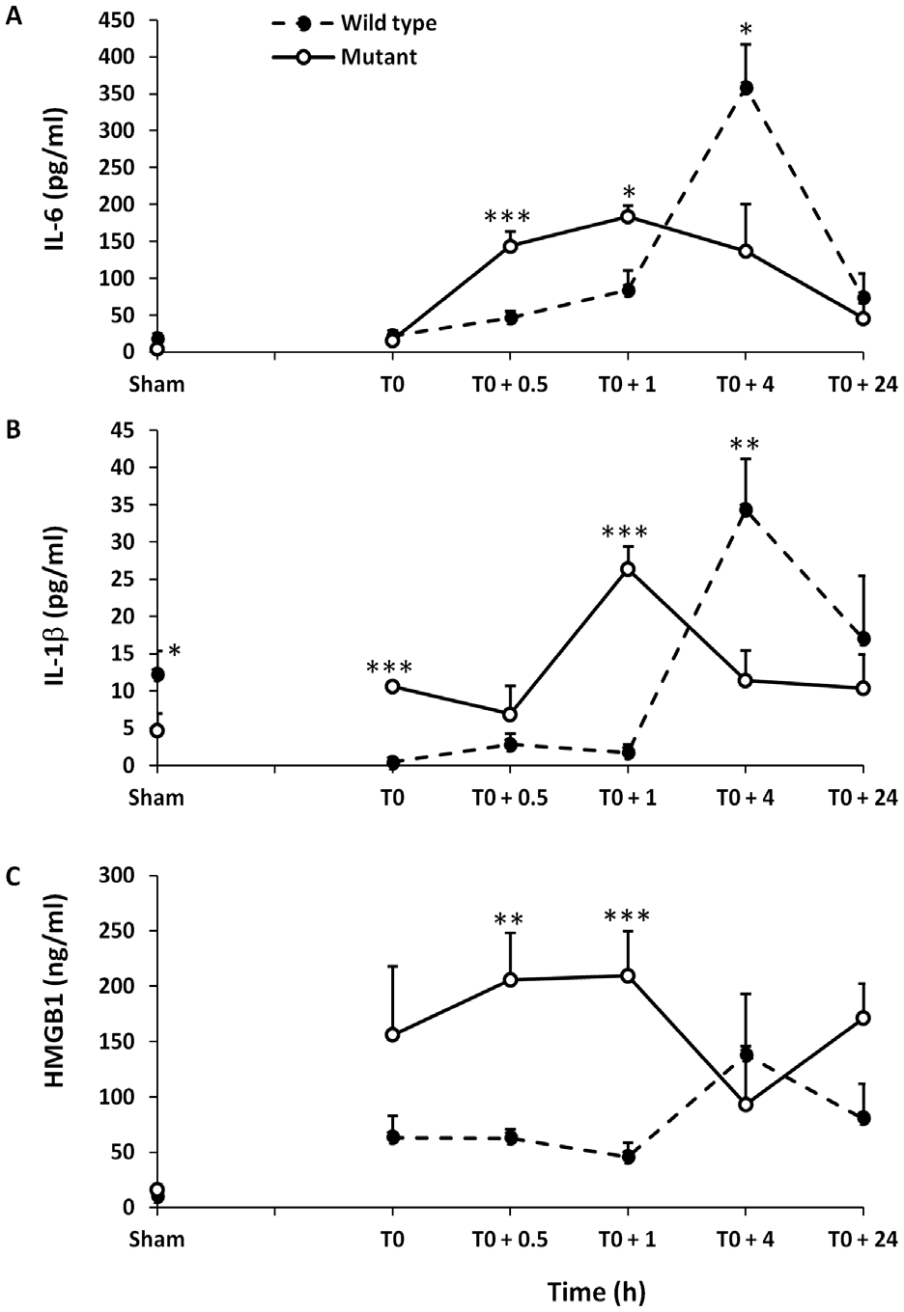
Comparison of plasma IL-6, IL-1β and HMGB1 levels in mutant and wild type mice. (**A**) Plasma levels (mean ± SD) of IL-6, (**B**) IL-1β and (**C**) HMGB1 are compared with mutant and wild type mice at the onset of heatstroke (T0) and at T0+0.5, +1, +4 and +24 hours post heatstroke. Plasma levels from sham-heated animals’ represent baseline. *P<0.05; **P<0.001; and ***P<0.0001 statistical significance at given time points between the two groups tested by the generalized estimating equations model.

We next investigated whether the increased vulnerability of mutant mice to heat stress was associated with the production of proinflammatory cytokines, particularly TNF-α, IL-1β, IL-6 and HMGB1. We measured the plasma levels of these cytokines in both strains prior to heat stress, at the onset of heatstroke and during the recovery period. Heatstroke induced a systemic inflammatory response characterized by significantly increased levels of IL-6, IL-1β and HMGB1, respectively from their baseline levels in both strains of mice. TNF-α was unchanged throughout the entire period of the study (data not shown). Importantly, however, the kinetics of cytokine release differed significantly between mutant and wild type mice. The mutant mice exhibited more inflammation in the first hour of heatstroke as evidenced by significant increase in IL-1β, IL-6 and HMGB1 levels at T0, +0.5 and +1 hour (Fig. 4). Conversely, the plasma IL-1β and IL-6 peaked later at T0+4 hours in wild type mice. Because, the baseline plasma IL-1β was different between the treatment groups, we adjusted for this difference using the generalized estimating equations. We found similar results before and after adjustment, thus suggesting that the difference in plasma IL-1β over time between mutant and wild type mice is independent of plasma IL-1β levels at baseline.

### Inhibition of HMGB1 Protects Mutant Mice Against Heatstroke-induced Lethality

**Figure 5:**
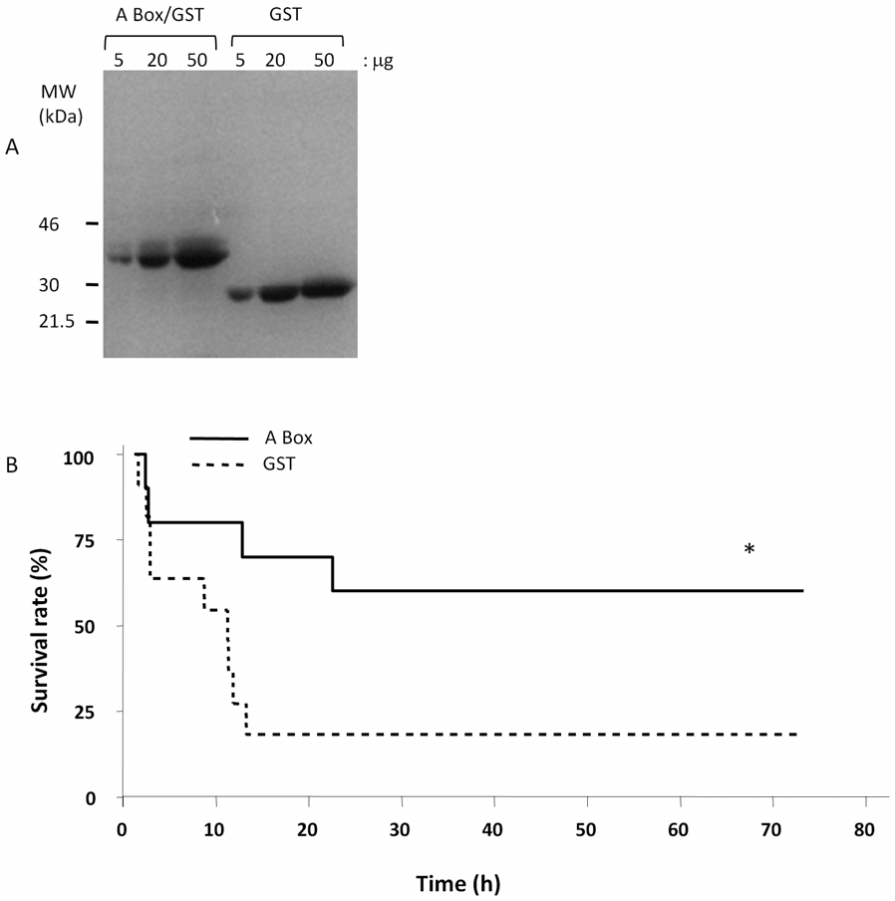
Inhibition of HMGB1 activity protects mutant mice against the lethal effects of heatstroke. (**A**) SDS-PAGE analysis of the recombinant A-Box and GST control proteins expressed and purified from *E. coli* is shown. Increasing amounts of purified proteins were resolved by SDS-PAGE and stained with Coomassie blue; (**B**) Mutant mice were pretreated (i.p. injection) with A Box 600 µg/mouse; or GST control and heatstroke was induced by passive exposure to environmental heat until the core temperature reached 42.7°C. Pretreatment with A Box before heat stress protected the mutant mice against the lethal effects of heatstroke. *P = 0.04 Kaplan-Meier log-rank testing between the two groups.

To determine whether HMGB1 played a role in the high lethality of mutant type mice to heat stress, we neutralized its activity by pre-treating these mice with purified A box protein, an antagonist of HMGB1 protein or GST as control and assessed the effect of heat stress on survival. A Box is a specific and powerful antagonist of endogenous HMGB1 that has been shown to reverse lethality of established sepsis in a mouse model [26]. Both proteins were overexpressed in *E. coli,* purified to near homogeneity (Fig. 5A) and administered i.p. immediately before heat stress. As shown in Fig. 5B, pretreatment with purified A box protein (600 µg/mouse) significantly protected the animals against the lethal effects of heat stress whereas no such effect was observed in GST-treated mice. The Kaplan-Meier curves show significant difference of survival (60% A Box vs. 18% GST protein, *P* = 0.04) between the two treatments.

## Discussion

The present study was designed to investigate whether the TLR4 and HMGB1 were implicated in the pathogenesis of heatstroke. Using a well-established murine model of heatstroke, we found that: (1) mutant mice were more susceptible to heat stress-induced histological liver damage and lethality than wild type mice; (2) HMGB1, the endogenous TLR4 ligand, was released very early at the onset of heatstroke in a sustained manner up to 24 h; and (3) inhibition of HMGB1 activity with DNA-binding A Box protected the mutant mice against the lethal effects of heatstroke. Taken together, these findings suggest that the TLR4 and HMGB1 are implicated in the pathogenesis of heatstroke, and thus targeting these mediators may have potential benefits.

TLR4 signaling pathway is activated during sterile insults such as hypoxia and ischemia and plays a critical role in cell survival, inflammation and tissue injury [15], [16], [17], [18], [21], [30]. The findings of the present study suggest for the first time that TLR4 plays a critical role during heat insult. Mice competent for TLR4 were more protected from tissue damage and death from heatstroke. This beneficial effect was associated with a delayed systemic inflammatory response as assessed by IL-1β and IL-6 levels and a concomitant reduced cell death and tissue infiltration with inflammatory cells. Hence, this suggests that a functional TLR4 signaling pathway is important for survival during heat stress. A similar protective role of TLR4 was demonstrated in experimental models of lethal oxidant or bleomycin-induced severe lung injury. In these studies [21], [30], the protective effect was attributed to TLR4 activation of cell survival and inflammatory genes via the NF-κB-dependant mechanisms, thereby reducing apoptotic cell death and impaired migration of inflammatory cells. Whether similar mechanisms are involved in protection from severe heat stress requires further studies.

Recovery from heatstroke has been associated with alteration of thermoregulation that manifests as hypothermia and/or rebound hyperthermia [25], [27], [31], [32]. The mechanisms and clinical significance of this thermoregulatory responses pattern are not yet fully understood. Early reports implicated heat-induced hypothalamic injury, but necropsies studies in large series of human with heatstroke failed to demonstrate any histological hypothalamic damage [31]. More recently, studies in mice show that a biphasic response of hypothermia followed by febrile response is constant during recovery from heatstroke, and suggest that this may be a regulated process through behavioral and autonomic thermal control [25], [27]. The present study shows that a comparable biphasic response pattern occurs in wild type mice but not in mutant. Indeed, mutant mice with deficient TLR 4 exhibited a sustained hypothermia and failed to mount a febrile response required to normalize the core temperature. Albeit indirect, this finding raises the hypothesis that TLR4 signaling may contribute to the thermoregulatory responses to severe heat stress that warrants further study. Our results demonstrate that the high lethality observed in mutant mice after heat stress is mediated to a greater extent by HMGB1. Both strains of mice displayed an early release of HMGB1 into the circulation, preceding that of IL-1β and IL6, which reached high levels, particularly in mutant mice. Pre-treatment with A-box, a competitive antagonist of HMGB1 protected the mutant type mice from lethality. These findings concur with those of a recent study in rat heatstroke model showing that plasma HMGB1 levels increase early in the course of heatstroke [23]. Pre-treatment with recombinant thrombomodulin which neutralizes HMGB1 activity through binding via thrombomodulin’s N-terminal lectin domain improved survival [23], [33]. Taken together, these observations suggest that HMGB1 is implicated in the pathogenesis of heatstroke.

HMGB1 exerts its cellular and biologic inflammatory responses by binding to three members of TLRs family namely TLR2, TLR4 and TLR9 as well as the receptor for advanced glycation end products (RAGE) [13], [19]. TLR4 is the primary receptor of endogenous HMGB1 in mediating cytokine release and tissue damage in various conditions, such as ischemia/reperfusion injury, hemorrhage and trauma and this mechanism of injury is attenuated or prevented by deficiency in TLR4 [15], [34], [35], [36]. However, our study showed that the HMGB1-induced tissue damage and death were independent of TLR4 signaling. The susceptibility of mutant mice to the lethal effect of heat was found to be associated with earlier and greater systemic inflammation together with increased infiltration of inflammatory cells into the liver and extensive hepatocytes necrosis culminating in death. In the absence of functional TLR4, one can speculate that HMGB1 may have engaged TLR2, TLR9 and/or RAGE to initiate the expression of IL-1β and IL-6, and other chemokines and adhesion molecules that facilitate the migration and accumulation of inflammatory cells [19]. Therefore, further studies will be required to establish the direct evidence that HMGB1 initiates the systemic inflammatory like-response to heat stress as well as to examine the expression and relative contribution of different TLRs implicated in the lethal effect of heatstroke. In conclusion, our study reveals for the first time that TLR4 is implicated in survival during heat stress. It also establishes that HMGB1 is an early mediator of inflammation in heatstroke which can contribute to tissue injury and lethality in the presence of defective TLR4 signaling. Further studies and better understanding of the role of endogenous danger molecules and TLR4 signaling pathway to heat stress should pave the way for novel therapeutic and preventive strategies of heatstroke.

### Perspectives and Significances

The current paradigm of heatstroke pathogenesis explains tissue injury and death in large part as a result of excessive inflammation induced by LPS leaking from damaged gastrointestinal mucosa [3], [37]. Our observation reveals that systemic inflammation, tissue injury and death occur in the absence of LPS signaling as C3H/HeJ mice belong to a strain that expresses a phenotype analogous to that of TLR4 knockout mice, thus hindering its signal transduction [24]. Further, it suggests that the presence of a functional TLR4, the main recognition receptor for LPS is important for survival to severe heat stress, thus suggesting that LPS does not play a significant pathogenic role. On the other hand, the findings that HMGB1 is released very early in the course of heatstroke and contributes to tissue injury and survival independent of TLR4 signaling mechanisms add further evidence to an emerging paradigm linking damage associated molecular patterns and innate immune response in the host defense against severe heat stress. Further understanding of this relationship may lead to novel preventive and therapeutic strategies.
